# Person-Centered Assessment: Evaluating Positive Psychosocial Measures in Dementia Research

**DOI:** 10.1093/geroni/igab046.1010

**Published:** 2021-12-17

**Authors:** Benjamin Mast, Sheila Molony

**Affiliations:** 1 University of louisville, louisville, Kentucky, United States; 2 Quinnipiac University, Hamden, Connecticut, United States

## Abstract

Person-centered principles continue to redefine the nature of dementia care, but less attention has been given to integration of person-centered principles into clinical assessment and dementia research. As a result, identification of deficits and cognitive impairment tends to dominate clinical and research efforts, whereas strengths and positive characteristics need more research. This paper examines existing positive psychosocial measures of psychological wellbeing, hope, spirituality, resilience, social relationship, dignity, and at-homeness. Many of these measures demonstrate strong psychometric properties and have been identified as promising outcome measures for strengths-based studies and approaches to care. This paper will evaluate the extent to which these measures used a person-centered approach to item development and testing, and whether item content is consistent with person-centered principles. Future directions for instrument development require greater inclusion of people living with dementia and family caregivers.

